# Systematic Literature Review of Functional Snacks: Bioactive Properties, Food Technologies, and Consumer Acceptance

**DOI:** 10.3390/foods15183345

**Published:** 2026-09-21

**Authors:** Linet Arvilla-Salas, Margarita L. Martinez-Fierro, Idalia Garza-Veloz, Maria del Refugio Rocha-Pizaña, Sodel Vazquez-Reyes

**Affiliations:** 1 Doctorado en Ciencias con Orientación en Medicina Molecular, Unidad Académica de Medicina Humana y C.S, Universidad Autónoma de Zacatecas, Carretera Zacatecas—Guadalajara Km 6, Ejido La Escondida, Zacatecas 98160, ZA, Mexico; nutrimentdmm@gmail.com (L.A.-S.); idaliagv@uaz.edu.mx (I.G.-V.); 2 Escuela de Ingeniería y Ciencias, Tecnológico de Monterrey, Av. Eugenio Garza Sada 2501, Monterrey 64849, NL, Mexico; mrochap@tec.mx

**Keywords:** functional snacks, anti-inflammatory, antioxidant, bioactive ingredients, food technologies, consumer acceptance

## Abstract

Functional foods containing antioxidants and anti-inflammatory compounds are a promising nutritional intervention. However, significant gaps remain in the literature on the development and evaluation of functional snacks for improving health outcomes. This systematic literature review (SLR) aimed to summarize the existing evidence on the development of enriched functional snacks containing bioactive ingredients, with a particular focus on their potential relevance to mental health. The review also examined food technologies, consumer acceptance, current knowledge gaps, and priorities for future research. The SLR was conducted in accordance with the PRISMA guidelines. PubMed, Scopus, and Web of Science were searched for studies on snacks and functional foods published between 2020 and 2025. Original studies evaluating functional snacks and/or bioactive compounds with neuroprotective, anti-inflammatory, or antioxidant properties were included. The initial search yielded 281,118 records. Following screening and quality assessment, 22 articles were selected through a three-author consensus process. Among the included studies, 12/22 focused on product development and antioxidant activity. Three studies included in vivo evaluations, while four included in vitro assessments of anti-inflammatory or antioxidant effects. Technologies such as extrusion, nanoencapsulation, and freeze-drying were employed to protect bioactive compounds and preserve bioactivity. Only two studies included sensory evaluation, with both reporting preliminary evidence of high acceptability. Overall, the SLR revealed limited evidence on functional snacks, particularly regarding their effects on mental health. Significant gaps remain in biological, neuroprotective, and sensory validation. Further studies are needed to establish the efficacy, biological effects, and consumer acceptability of enriched functional snacks, as well as consumer acceptability across diverse, relevant target populations.

## 1. Introduction

In recent years, functional foods have become one of the most innovative areas of nutritional science. This field incorporates bioactive compounds with antioxidant, anti-inflammatory, and physiological process-modulating properties that may provide health benefits beyond basic nutrition [1]. Functional foods can modulate one or more physiological functions, thereby contributing to health benefits. The term was coined in the 1980s, and since then, the development and regulation of functional foods have been addressed by international organizations such as the Commission on Food, Nutrition, and Development (CPDEX), the World Health Organization (WHO), the Food and Agriculture Organization of the United Nations (FAO), and the International Alliance of Dietary/Food Supplement Associations (IADSA) [2]. Although functional foods are regulated, most countries do not legally recognize them, leaving no unified legal definition [3]. They have recently been defined as natural or industrially processed foods that when consumed regularly as part of a varied diet and in effective amounts, may provide health benefits beyond basic nutrition [2].

Numerous studies have shown that consuming certain nutrients and following healthy dietary patterns, such as the Mediterranean diet, which emphasizes a high intake of fruits, vegetables, seeds, and legumes [4,5,6], is associated with a lower risk of anxiety and depression symptoms. This has sparked interest in exploring nutritional interventions as a complementary strategy for improving mental health [1,5,7]. Diet is one of several external factors that influence gut microbiota composition and may affect communication along the gut–brain axis. In this context, functional foods enriched with bioactive compounds may represent a potential nutritional strategy relevant to mental health, exerting neuroprotective effects by mitigating oxidative stress and neuroinflammation. This may occur through direct molecular interactions in the central nervous system that regulate mood, supporting their potential relevance as a nutritional strategy for mental health [8,9].

Numerous international studies have documented a significant association between dietary patterns and mental health among college students [8,9,10,11]. For example, in the United States, high levels of anxiety and depression among college students have been linked to a lower intake of fruits and vegetables, as well as higher added sugar consumption, particularly among male students [12]. Similarly, in Iran, adherence to plant-based dietary patterns was inversely associated with depressive symptoms, though no significant association was found with anxiety levels [13]. During the 2020–2021 lockdown in India, college students without depressive or anxiety symptoms were more likely to maintain a higher-quality diet, suggesting a bidirectional relationship between diet and mental health [8]. Furthermore, a binational study of students in England and Turkey found that those with greater nutritional knowledge and higher adherence to the Mediterranean diet reported lower levels of anxiety and depression [4].

Despite advances in research, there is still a significant lack of information in the scientific literature on the use of functional foods as a complementary therapy for mental health disorders. To date, no study has examined the effect of technological processing on the stability and retention of bioactive compounds in food, particularly in snack products. This is important because processing methods can influence the efficacy and stability of bioactive compounds, thereby potentially affecting the biological activity and health-related effects of the resulting food products. While most studies focus on the general effects of individual nutrients (e.g., omega-3 fatty acids, polyphenols, and probiotics) or on nutraceutical foods such as dietary supplements [1,14,15], an integrative analysis linking the technological design of functional foods with clinical evidence in mental health is lacking. Additionally, the literature on functional foods intended to modulate mood- and cognition-related processes has several gaps. (a) No systematic reviews have been published that specifically address the role of functional foods as a treatment strategy for patients with mental health disorders. Evidence for other diseases is also very limited. (b) Findings are often heterogeneous and do not consider how individual variables, such as disorder severity, sex, or lifestyle habits, might affect the response to these foods. (c) Studies tend to focus on isolated components without evaluating the interactions between bioactive compounds within designed food matrices. (d) Few studies address sensory acceptability and adherence to functional food consumption. These are key factors for their implementation in clinical practice.

Based on the above, this systematic literature review (SLR) summarizes the existing evidence on the development of enriched functional snacks containing bioactive ingredients with anti-inflammatory and antioxidant properties, with particular emphasis on their potential relevance to mental health, while examining food technologies, consumer acceptance, current knowledge gaps, and priorities for future research. The objective was to lay the groundwork for food innovation and support the development of new nutritional strategies for mental health.

## 2. Materials and Methods

This SLR was conducted in accordance with the Preferred Reporting Items for Systematic Reviews and Meta-Analyses (PRISMA) guidelines [16]. The guidelines for conducting systematic literature reviews, as established by Kitchenham et al. [17] were also followed. Adhering to these guidelines ensured methodological transparency and minimized the risk of bias. The process began with identifying the research questions (Section 2.1), defining the search strategy (Section 2.2), establishing the inclusion and exclusion criteria (Section 2.3), assessing the quality of the included articles (Section 2.4), and extracting the relevant data (Section 3). We summarized the results of the individual studies using descriptive tables and narrative synthesis. A team of three authors monitored, validated, and approved all critical stages of the systematic review. To ensure methodological robustness, independent double screening was performed.

### 2.1. Research Questions

Considering the limitations identified in the previous literature reviews, we developed four research questions for further study. The questions were formulated using the PICOC principle (Patient/Population, Intervention, Comparison, Outcomes, and Context). The research questions are as follows:

(1) What types of functional snacks with psychoactive, anti-inflammatory, or antioxidant properties have been developed and reported in the scientific literature?
(2) Which bioactive ingredients are most commonly used in the formulation of these functional snacks (such as polyphenols, flavonoids, adaptogens, probiotics, and omega-3 fatty acids)?
(3) What food technologies (e.g., extrusion, baking, freeze-drying, and encapsulation) are most effective in preserving the functional properties of bioactive ingredients in snacks?
(4) What is the reported consumer acceptance of functional snacks in terms of sensory properties and market attractiveness?

### 2.2. Search Process

This SLR was conducted in accordance with the Preferred Reporting Items for Systematic Reviews and Meta-Analyses Statement (PRISMA) [16]. We examined scientific articles written in English and Spanish that were published between 2020 and 2025 and indexed in PubMed, Scopus, and Web of Science. To develop a focused search strategy, we selected keywords related to functional snacks and their potential relevance to mental health. Keywords included functional snacks, anti-inflammatory, antioxidant, bioactive ingredients, food, technologies, and consumer acceptance.

We created search strings using Boolean operators. The search was filtered to the Title/Abstract fields and limited to the period from 2020 to 2025. We also limited the search to free full-text articles in English or Spanish and to studies involving humans and other animals. Preprints were excluded. We applied combinations of keywords using the Boolean operators “AND” and “OR”, as well as quotation marks. The same search strings were used across all databases and were structured as follows:

- First, the terms for functional snacks were combined as follows: “functional snacks psychoactive effect” OR “anti-inflammatory” OR antioxidant.
- Second, regarding the terms for bioactive compounds, the search query was combined as follows: “functional snacks” AND (“bioactive compounds” OR “psychotropic effects” OR “anti-inflammatory activity” OR “antioxidant capacity”); “bioactive ingredients” AND (polyphenols OR flavonoids OR adaptogens OR probiotics OR “omega-3”).
- Third, for food technologies, the terms were combined as follows: (“food technology” OR “food technologies”) AND (extrusion OR baking OR freeze-drying” OR “encapsulation”) AND (“functional properties” OR “preservation of functional compounds” OR “bioactive retention”) AND (“bioactive compounds” OR “psychotropic effects” OR “anti-inflammatory activity” OR “antioxidant capacity”).
- Finally, the search string for consumer acceptance was combined as follows: (“consumer acceptance” OR “consumer perception” OR “consumer preference”) AND (“sensory properties” OR “sensory evaluation” OR “organoleptic properties”) AND (“market attractiveness” OR “market potential” OR “purchase intention”) AND “functional snacks”.

The entire search and eligibility process was created using an Excel document (https://docs.google.com/spreadsheets/d/1dTE-EvebbYSSYIMd36hA-3nv15GeHXAx/edit?gid=1929611959#gid=1929611959, accessed on 14 May 2026).

### 2.3. Eligibility Criteria

**Figure 1:**
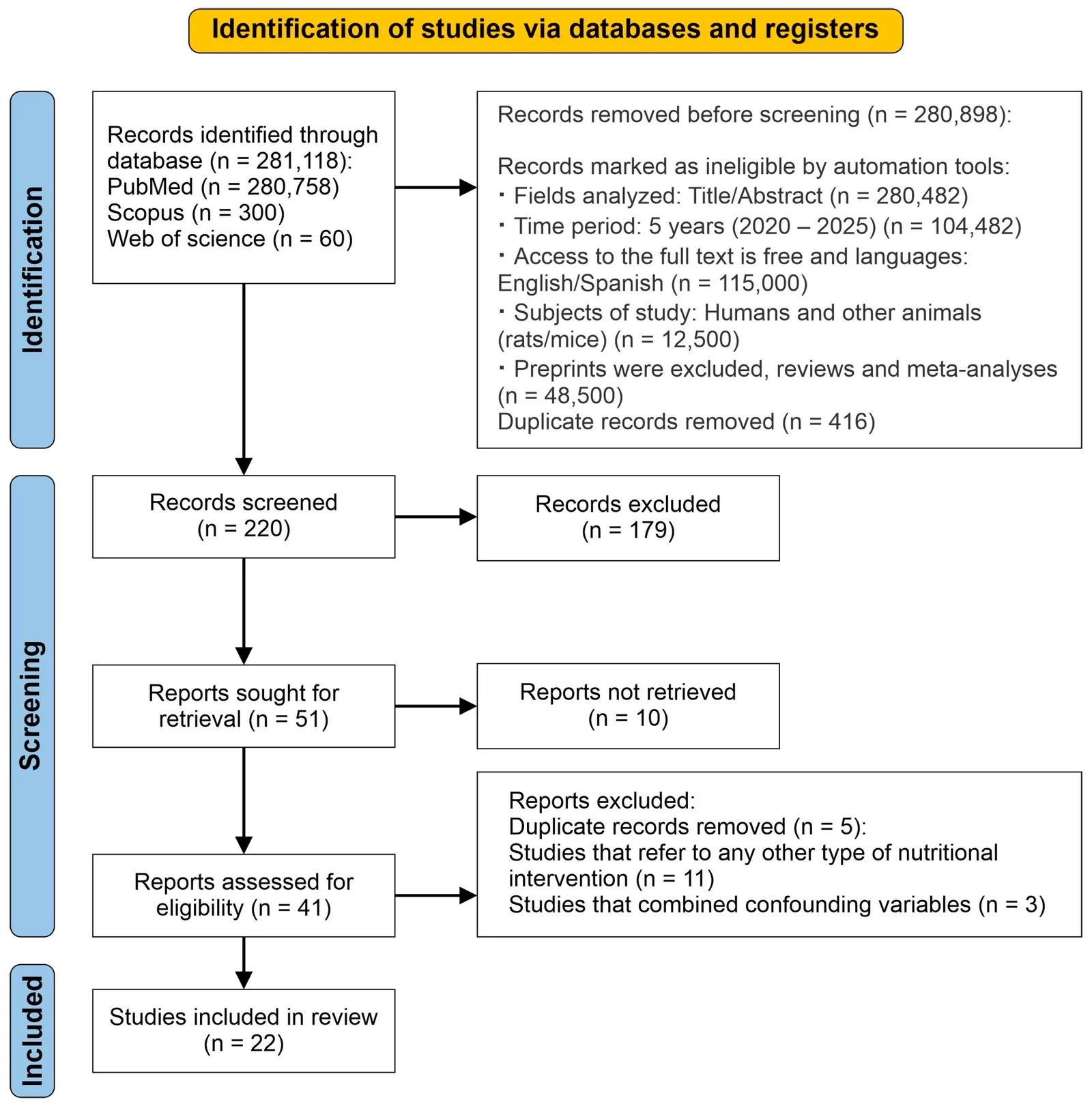
PRISMA flowchart of the study selection process. This diagram summarizes the methodology used to select studies for this systematic review, from the initial identification of relevant literature in databases such as PubMed, Scopus, and Web of Science to the final selection of studies. The selection methods at each stage are reported, including automatic exclusion based on criteria such as search fields, time period, full-text access, language, and study population, as well as the exclusion of preprints, literature reviews, and meta-analyses. The diagram also details the specific exclusion criteria applied during the full-text analysis stage, such as duplicate records, nutritional interventions outside the study scope, and studies with confounding variables. Blue boxes indicate the stages of the process, while white boxes illustrate the selection processes and exclusion criteria at each stage.

We analyzed the studies selected from the specified databases in several stages. First, titles and abstracts were screened, and records were excluded using automated filters on the search platforms, as shown in Figure 1. These automated filters applied the following criteria: publication period from 2020 to 2025 (*n* = 104,482); full-text availability and language restricted to English and Spanish (*n* = 115,000); study subjects including humans and animal models, such as rats and mice (*n* = 12,500); and exclusion by document type, omitting preprints, reviews, and meta-analyses (*n* = 48,500). Finally, we removed 416 duplicate records. We then carefully reviewed the selected articles and excluded those deemed irrelevant to this study in accordance with the selection criteria. The database search and selection process adhered to the PRISMA flowchart, covering the identification, screening, eligibility assessment, and final inclusion phases. One author conducted the search and selection process, with support from two others, who reviewed each phase.

#### Inclusion and Exclusion Criteria

Table 1 presents out the inclusion and exclusion criteria used to select the studies. A summary of the selected studies is also provided, including study type, publication period, language, thematic relevance, properties assessed, and models or study populations used.

Furthermore, when selecting the studies, we defined a “functional snack” as a small portion consumed between main meals that not only satisfies hunger but also provides a specific health benefit, such as boosting immunity, improving brain function, or regulating metabolism, due to the presence of bioactive ingredients and/or added nutrients [18]. Based on the NOVA classification system, we also included processed and ultra-processed snacks [19].

It is also important to note how the heterogeneity of terms related to “mental health” was addressed. Terms such as “psychoactive”, “neuroprotective”, “anti-inflammatory”, and “antioxidant” were included. We chose these terms to focus on bioactive compounds that influence neuromodulatory and neuroprotective mechanisms or the gut–brain axis.

Due to the very small number of functional snacks identified, we included six additional studies on “functional foods” (not snacks). The inclusion criteria for the core and expanded literature were aligned with the following: study type (functional snacks/foods); time period; language; relevance; subjects/models and properties studied.

### 2.4. Data Extraction Strategy

The article selection process consisted of the following stages. First, one author independently reviewed the titles, abstracts, and keywords and excluded studies that did not meet the review’s eligibility criteria. Duplicate articles were removed. During this phase, we deemed records ineligible if they did not evaluate functional snacks or did not report antioxidant, anti-inflammatory, or neuroprotective effects. Next, we applied the inclusion and exclusion criteria (see Table 1) to select the studies that best fit the research questions. Two additional authors independently reviewed and approved all stages. Finally, we assessed the selected articles using 12 quality questions (see Tables S7–S9 in the Supplementary Materials). We adapted these questions from the guidelines established by Kitchenham et al. [17] to minimize bias and maximize the methodological quality of this review. In addition, one author carefully read the full texts to extract and synthesize the data. Data were extracted using an Excel spreadsheet (Microsoft Excel, Microsoft Corporation, Redmond, WA, USA) to ensure the thorough and systematic extraction of study characteristics (https://docs.google.com/spreadsheets/d/1dTE-EvebbYSSYIMd36hA-3nv15GeHXAx/edit?gid=1929611959#gid=1929611959, accessed on 14 May 2026). The entire data extraction process involved independent dual screening.

## 3. Results

### 3.1. Study Selection

Figure 1 summarizes the study selection process. A total of 281,118 articles were identified via database searches (PubMed: *n* = 280,758; Scopus: *n* = 300; Web of Science: *n* = 60). After removing duplicates and irrelevant records, 220 studies remained. Title and abstract screening yielded 41 potentially relevant studies. After full-text assessment against the predefined inclusion and exclusion criteria (see Table 1), we excluded 19 studies. Finally, 22 studies were included. Eight of these were on functional snacks, six were on the production of functional foods, and eight were on food processing technologies designed to preserve bioactive compounds. The included studies were also assessed using a 12-item questionnaire covering study design, conduct, analysis, and conclusions.

### 3.2. Characteristics of the Studies

The literature search identified only a limited number of studies focusing on the development of functional snacks (see Table 2). The most common snack types were fried extruded foods [20], beverages [21], bread [22], bars [23,24], chocolates [11,25], and cookies [26]. Many of these studies (7/22) were limited to the “development and characterization” phase. Only one study [11] advanced to in vivo evaluation, demonstrating that chocolate enriched with polyunsaturated fatty acids (PUFAs) and probiotics increased the number of neurons in the CA1 and CA3 regions of the rat hippocampus. The most frequently evaluated bioactive components were polyphenols (e.g., resveratrol, gallic acid, and flavonoids/anthocyanins) and essential macromolecules (e.g., proteins and fiber) as well as specific ingredients such as omega-3 fatty acids and probiotics.

**Figure 2:**
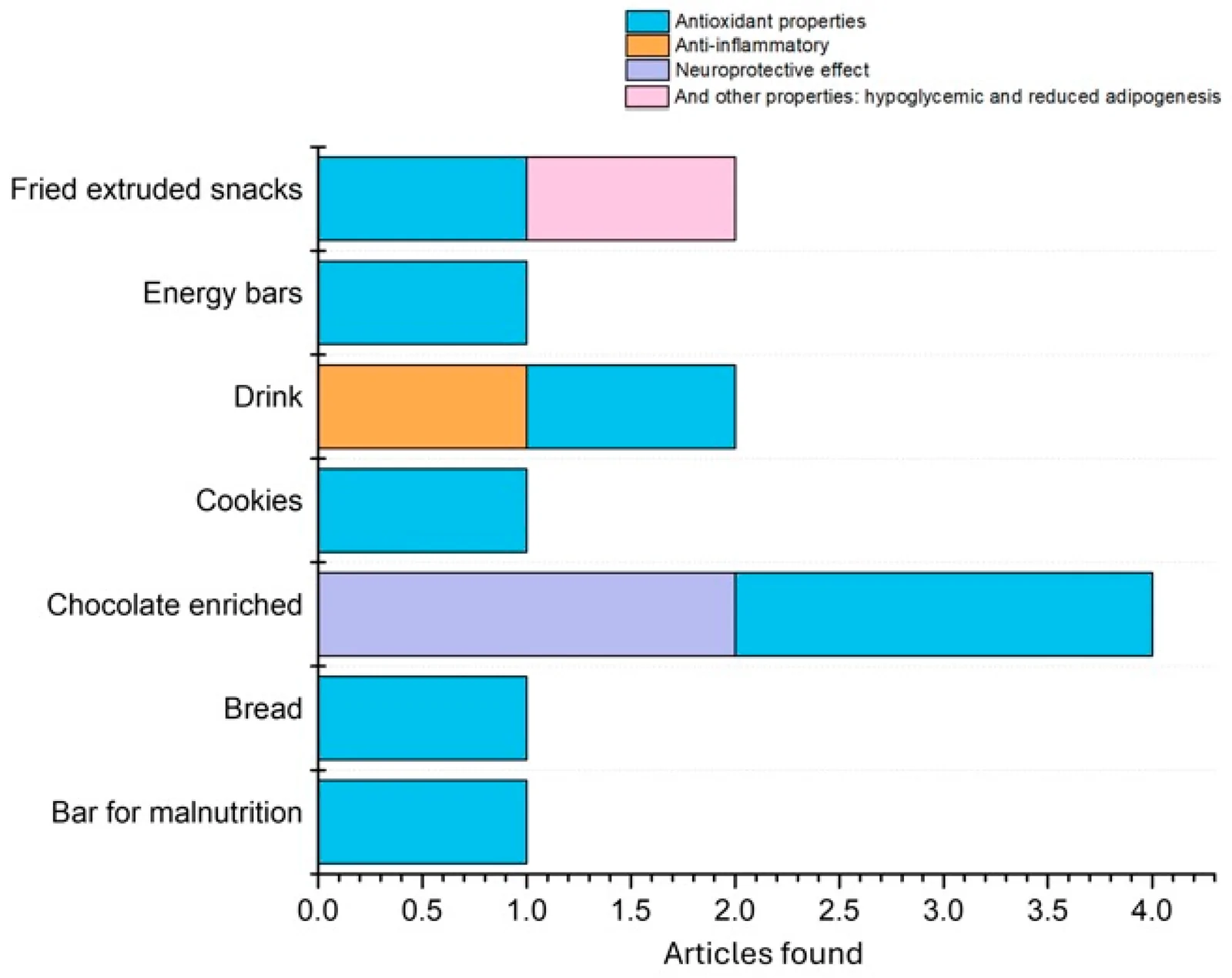
Distribution of the included articles by type of snack developed and bioactive properties evaluated. This figure visually summarizes the distribution of the included studies by snack type. The studies were classified to enable clear comparison of the main functional properties related to mental health, particularly antioxidant and anti-inflammatory properties. The colored segments within the bars identify the specific biological activities evaluated. The blue blocks represent antioxidant properties, the orange blocks indicate anti-inflammatory effects, the lilac blocks highlight neuroprotective effects, and the pink blocks denote other properties reported in the studies, such as hypoglycemic activity and reduced adipogenesis.

Of the functional properties evaluated, antioxidant activity was the most prevalent, being present in most types of processed snacks. Some studies also investigated other effects. For instance, one study on a guava beverage reported antioxidant and anti-inflammatory properties [21], while another study on snacks containing non-encapsulated resveratrol demonstrated significantly greater hypoglycemic effects and reduced adipogenesis. Additionally, an extruded snack with hypoglycemic effects that reduced adipogenesis was found to have antioxidant and anti-inflammatory properties, as was a beverage [21]. Enriched chocolate was found to have neuroprotective effects, increasing the number of neurons in the CA1 and CA3 regions of the hippocampus [11]. Studies that did not mention any properties beyond the neuroprotective, anti-inflammatory, and antioxidant effects were considered to lack other properties, as shown in Figure 2 [11,20].

**Figure 3:**
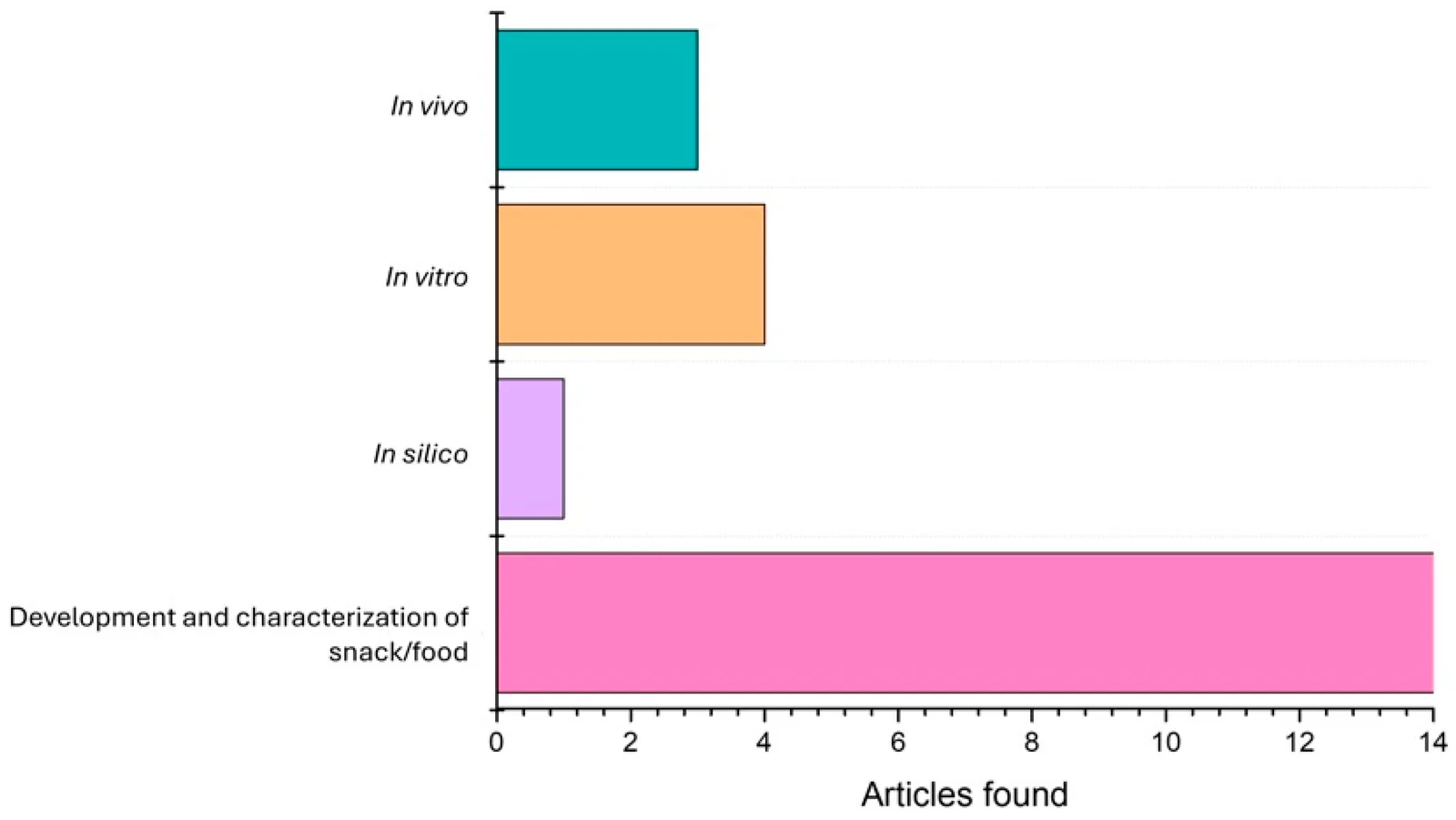
Classification of the selected articles by study type. This horizontal bar chart visually summarizes the distribution of 14 out of the 22 studies included in this SLR (8 snacks and 6 functional foods); the remaining 8 studies on food processing technologies are presented in Table 4. The studies are classified by study type: snack/food development and characterization; in silico, in vitro, and in vivo studies. Some studies are included in more than one category because they underwent multiple evaluation stages; therefore, the number of bars exceeds the number of articles represented. The *x*-axis shows the number of articles found. The chart distinguishes among four main research categories, separating biological models from product formulation phases. Different colors identify each category: teal represents in vivo studies, orange represents in vitro methodologies, purple represents in silico computational approaches, and pink represents articles focused on the development and characterization of snacks/foods.

As shown in Figure 3, the scarcity of studies with in vitro or in vivo evidence, together with the absence of clinical evaluations, indicates that research has focused primarily on formulation and technological development. The included studies focused more on formulation and technological development, leaving aside the importance of biological evidence (in vitro and in vivo studies, as well as clinical trials) for evaluating functional effects in this context. Most of the research fell under the category “Development and characterization of snacks/foods”, with 14 articles [11,20,21,22,23,24,25,26,27,28,29,30,31,32] considering functional foods in additional studies. This suggests that current scientific efforts are primarily focused on the design, optimization, and physicochemical evaluation of developed functional snacks/foods. In vitro studies were the most common biological evaluation method, accounting for four articles [27,28,29,30]. Three articles used in vivo models to validate health properties or nutritional effects in living organisms, closely following in vitro studies [11,27,30]. In silico analysis was the least explored approach, with only one article employing computational models or bioinformatics tools [30]. Notably, clinical trials were not explored, as none were found among the studies included in this review.

In addition, given the limited number of studies on functional snacks, we included six additional studies on the development of functional foods enriched with bioactive compounds (see Table 3). These functional foods included habanero chili by-products [27], lupin protein hydrolysates [28], resveratrol [29], cashew leaf extract [30], blueberries [31], and banana flour [32]. Four of these studies confirmed anti-inflammatory and antioxidant effects using in vitro [27,28,29], in vitro and in vivo [27,30], and in silico [30] methodologies. For example, lupin hydrolysates and resveratrol showed anti-inflammatory effects in cell models [28,29]. The remaining two studies focused on the “characterization and development” of food matrices, emphasizing compound retention and improved quality parameters [31,32].

### 3.3. Studies That Used Food Technology to Preserve Functional Properties

The food processing techniques most often reported in the included studies as preserving functional properties were extrusion, nanoencapsulation, drying, and fluid extraction. In food technology, extrusion is used to produce enriched snack products, such as functional cookies containing sorghum or millet protein nanoparticles [31,33], as well as products containing rice and pea proteins combined with cranberry powder [31]. The results showed that extrusion could retain bioactive compounds, such as crocin (58–77% retention) and antihypertensive properties via ACE inhibition (40.53–53.36%, *p* < 0.05) [33]. It also influences the physicochemical properties by reducing the outlet temperature (OAT: 60 °C; IAT: 150 °C) and the expansion ratio as the moisture content increased [26]. SDYP was most effective for functional cookies, significantly enhancing their sensory and biochemical characteristics (*p* < 0.05). Conversely, resveratrol was nanoencapsulated in starch nanoparticles, using three different starch sources, water chestnut, lotus root, and horse chestnut, to protect it from gastric conditions and enhance its bioavailability and bioactivity post-digestion [20]. The authors prepared the nanocapsules using a sonication method that was both safe and environmentally friendly [20]. The capsules were characterized using attenuated total reflectance-Fourier transform infrared spectroscopy (ATR-FTIR), scanning electron microscopy (SEM), X-ray diffraction (XRD), and differential scanning calorimetry (DSC).

This strategy was crucial for protecting the sensitive compounds, resulting in higher retention rates after extrusion (43–53%) than those observed with free resveratrol (5.42%). In turn, this enhanced the snack’s hypoglycemic effects (23.23% to 63.23%) and reduced adipogenesis (39.46%, 44.46%, and 24.86% for HRP, WRP, and LRP, respectively). In its free form, resveratrol showed significantly lower inhibition (18.93%) (*p* < 0.05). Other examples include broccoli sprout methanol extract (BSE) nanoliposomes co-encapsulated in basil seed gum (BSG) broccoli sprout methanol extract (BSE) [34], and the use of β-lactoglobulin nanostructures to encapsulate hydrophobic molecules such as quercetin (96.5% ± 0.08, *p* < 0.05) and rutin (89.04% ± 0.17, *p* < 0.05) with high efficiency (up to 96.5%) [35]. Thermal processing technologies such as advanced drying and extraction methods also played a key role, as explained below.

Researchers evaluated various drying techniques for producing functional banana flours, including freeze-drying (FD), cabinet drying (CD), microwave drying (MD), and forced-air drying (FAD) [32]. The results showed that freeze-drying was the most effective method, as it best preserved the flour’s functional properties of the flour best and improved its flow characteristics. Freeze-dried Mehersagar flour exhibited the best functional properties, demonstrating good flowability and cohesion. Significant differences in the physicochemical parameters of the flours were also observed (*p* < 0.05); see Table 4 for details.

**Table 4:** Characteristics of studies using food technology to preserve functional properties.

Authors and Year	Food Technology Used to Preserve Functional Properties	Food or Functional Snack	Key Findings
Ismail M. et al., 2025 [36]	Vacuum osmotic dehydration (VOD) was used to produce orange slices.	Pomegranate juice was used to produce orange slices	The HA-RFD technique preserved the functional properties of the fortified dried fruits while reducing the drying time by 62%, (from 1110 min with HAD to 420 min). The HA-RFD technique produced fruits with a higher TPC of 1496 mg GAE/100 g dry weight, a TFC of 415 mg rutin/100 g dry weight, and an antioxidant activity of 90%.
Peña-Vázquez et al., 2025 [37]	The antioxidant and anti-inflammatory properties of the lipids were determined using subcritical and supercritical fluid extraction with carbon dioxide (SCE-CO_2_ and SFE-CO_2_), with and without enzymatic pretreatment.	OFI seed oil from the Mexican varieties “Villanueva” and “Rojo Vigor” was evaluated	Supercritical CO_2_ extraction improved the recovery of unsaturated fatty acids and demonstrated higher antioxidant (67.97 ± 0.45%). There were no statistically significant differences compared with maceration (26.67 ± 5.70%). Furthermore, the extract exhibited 45% anti-inflammatory activity (*p* < 0.0296).
Jhan et al., 2023 [33]	Fabrication of protein-crocin nanocomplex. Contains crocin in sorghum protein nanoparticles (SPCS), pearl millet protein nanoparticles (PPCS) and foxtail millet protein nanoparticles (FPCS).	Millet flour enriched with a crocin-protein nanocomplex	The crocin content was adequately preserved in the millet flour snacks (58.08% and 77.67%). These snacks exhibited antioxidant activity and protective effects against DNA damage, as well as hypoglycemic activity through α-amylase inhibition (49.35–57.87%) and antihypertensive properties through ACE inhibition (40.53– 53.36%, *p* < 0.05).
Ahmad and Gani, 2021 [20]	Fried extruded food with nanoencapsulated resveratrol.	Functional snacks with resveratrol	After extrusion, 43–53% and 5.42% of the resveratrol were retained in snacks containing encapsulated and free resveratrol, respectively. The former also exhibited greater hypoglycemic effects (23.23–63.23%) and reduced adipogenesis (39.46%, 44.46%, and 24.86% for HRP, WRP, and LRP, respectively). In its free form, resveratrol exhibited significantly (*p* < 0.05) lower inhibition (18.93%). Nanoencapsulated resveratrol exhibited enhanced antioxidant activity. The highest inhibition value was observed for LRP (61.85%), while the lowest was observed for FRP (40.78%). Nanoencapsulated resveratrol, through its antioxidant properties, positively affected the oxidative stability of the extrudate.
Alharaty and Ramaswamy, 2025 [31]	Fried snacks made with vegetable protein enriched by low-temperature extrusion and air drying.	Extruded matrix enriched with RP, PP, and natural cranberry powder	Compared with extrusion with a lower moisture content, extrusion with a higher moisture content (40% MC) reduced the outlet temperature by 61.97 °C to 55 °C (*p* < 0.05), the expansion ratio from 1.26 to 1 (*p* < 0.05), and the rehydration ratio from 78.70% to 31.90%.
Azarashkan et al., 2022 [34]	The antioxidant and antibacterial activity (AOA and ABA) of broccoli sprout extract (BSE) nanoliposomes co-encapsulated in basil seed gum (BSG).	Advanced formulation of broccoli sprout extract	Nanoliposomes and nanocapsules loaded with BSE showed potential for use as natural antioxidants and preservatives in food products. DPPH activity was similar between free and encapsulated BSE, ranging from 74.93% to 75.29%, and the difference was not statistically significant (*p* > 0.05).
D’Onofre Couto et al., 2021 [35]	Improved production of β-lactoglobulin (β-lg) nanostructures as vehicles for bioactive molecules.	β-lg nanostructures	The encapsulation efficiencies were 96.50 ± 0.08% for quercetin, 89.04 ± 0.17% for rutin, 67.78 ± 5.03% for naringin, and 36.39% for vitamin B2.
Mutavski et al., 2025 [38]	Encapsulation of black elderberry pomace powders obtained by spray drying with maltodextrin and gum Arabic.	Encapsulation of black elderberry pomace powders	The stability and bioavailability of elderberry extract were significantly improved (*p* < 0.05) through encapsulation, which effectively retained bioactive compounds.
Ramu Ganesan et al., 2023 [39]	The rheological properties of doughs containing 50% brewer’s spent grain (BSG) derived from rye-based beer (RBSG) and barley-based beer (BBSG).	Doughs containing 50 per cent brewer’s spent grain (BSG)	The rheological properties of doughs containing 50% brewer’s spent grain (BSG) derived from rye-based (RBSG) and barley-based (BBSG) beers were investigated. The dynamic rheological moduli (G′, G″ and G*) and complex viscosity showed highly significant positive correlations with hardness and gumminess (r = 0.76–0.97, *p* > 0.01). A highly significant correlation was also found between gumminess and resilience, with associations involving G′, G″, G*, and complex viscosity (r = 0.76–0.90, *p* > 0.01).
Ali et al., 2023 [26]	Spray-dried yogurt powder (SDYP) for cookie preparation.	Functional cookie	A combination of temperatures (OAT: 60 °C; IAT: 150 °C) was found to maximize the survival of the inoculated cultures, while SDYP was found to be most effective for functional cookies, significantly enhancing their sensory and biochemical characteristics (*p* < 0.05). The maximum moisture content was 5.21 g/100 g, the maximum protein content was 16.22 g/100 g, and the lowest fat content was 20.98 ± 0.01 g/100 g.
Alam et al., 2023 [32]	Flour production by freeze-drying (LF), cabinet drying (CD), microwave oven drying (MOD) and forced air oven drying (FOD).	Banana flours	The freeze-dried Mehersagar flour demonstrated the best functional properties, exhibiting good flowability and cohesion. The physicochemical parameters of the flours also showed significant differences (*p* < 0.05) in lightness (50.51 ± 1.49–72.21 ± 1.05), moisture content (3.96 ± 0.09–7.74 ± 0.13%), protein (2.72 ± 0.07–3.93 ± 0.06%), crude fat (0.11 ± 0.01–0.36 ± 0.04%), crude fiber (0.64 ± 0.03–1.22 ± 0.03%), carbohydrate content (84.15 ± 0.24–88.26 ± 0.15%) and energy content (354.25 ± 0.57–370.02 ± 0.39 kcal/g). Total flavonoid content (21.44 ± 0.04–34.34 ± 0.03 mg QE/100 g) and total phenolic content (29.91 ± 0.01–71.46 ± 0.03 mg GAE/100 g) also differed significantly among the flours
Sotelo-Díaz et al., 2023 [40]	Extruded blends of corn and cowpea flours	Maize and cowpea flours	The SWE of the mixtures increased significantly (*p* < 0.05) following extrusion and drying, and the resulting product could be used as a protein source when rehydrated. Furthermore, storing the processed samples as pellets prevented them from becoming damp. This format was less hygroscopic and occupied less storage volume for the same mass.

VOD: Vacuum osmotic dehydration, HA-RFD: Radiofrequency-assisted hot air drying, HAD: Hot air dehydration, SCE-CO_2_: Subcritical fluid extraction with carbon dioxide (CO_2_), SFE-CO_2_: Supercritical fluid extraction with carbon dioxide (CO_2_), OFI: Opuntia ficus-indica, SPCS: Sorghum protein crocin system/complex, PPCS: Pearl millet protein crocin system/complex, FPCS: Foxtail millet crocin protein system/complex, RP: Rice protein, PP: Pea protein, MC: Moisture content, AOA: Antioxidant activity, ABA: Antibacterial activity, β-lg: β-Lactoglobulin, BSG: Brewers’ spent grain, BBSG: Rye brewers’ spent grain, SDYP: Spray-dried yogurt power, OAT: Outlet air temperature, IAT: Inlet air temperature, LF: Lyophilized, CD: Cabinet drying, MOD: Microwave oven drying and FOD: Forced air oven drying; TPC: Total phenolic content, GAE: Gallic acid equivalent, TFC: Total flavonoid content, SWE: Swelling index. G′: The elastic modulus, G′′: Viscous modulus, G*: Complex modulus.

Significant differences were also observed in the physicochemical parameters of the flours, including lightness (L*), moisture content, protein content, crude fat content, crude fiber content, carbohydrate content, and energy content (*p* < 0.05). Total flavonoid content ranged from 21.44 ± 0.04–34.34 ± 0.03 mg QE/100 g, and total phenolic content ranged from 29.91 ± 0.01–71.46 ± 0.03 mg GAE/100 g. Conversely, vacuum-assisted radiofrequency freeze-drying (HA-RFD), combined with vacuum osmotic dehydration (VOD), was used to process orange slices, preserving their functional properties while reducing drying time by 62% [36].

In contrast, advanced fluid extraction methods, including subcritical and supercritical carbon dioxide extraction (SCE-CO_2_ and SFE-CO_2_), were employed to obtain functional lipids from the seeds of Mexican *Opuntia ficus-indica* (OFI) plants of the Villanueva and Rojo Vigor varieties [37]. These methods not only improved the extraction efficiency of unsaturated fatty acids but also yielded extracts with greater antioxidant potential (68%) and anti-inflammatory activity (45%). Other technologies included spray drying to produce yogurt powder for cookies, which achieved the maximum survival of inoculated cultures at specific temperatures [26], and evaluating the rheological properties of doughs enriched with rye and barley brewer’s spent grains for the 3D printing of functional foods [39].

### 3.4. Studies That Included Sensory Evaluation

Of the 22 articles selected for review, only two incorporated a sensory evaluation into their research. Notably, both studies focused on developing and characterizing snack products with functional properties (see Table 5). The aim of these studies was to determine the acceptance and organoleptic characteristics of innovative products incorporating functional ingredients. One study evaluated fried extruded snacks with nanoencapsulated/free resveratrol using a semi-trained panel (*n* = 15–20). A key finding was that adding resveratrol (free or encapsulated) did not affect the product’s sensory properties. The maximum flavor and overall acceptability values were 7.51 ± 0.85 and 7.45 ± 0.44, respectively, on a 9-point scale [20]. All samples received the same level of acceptance. Another study evaluated energy bars using 50 untrained panelists. The sensory evaluation yielded average attribute scores ranging from 3.4 to 4.1 on a 0–5 scale. Furthermore, the panelists reported no perceptible differences between the various samples [23].

The evidence indicates a significant gap in the current research on the development, characterization, and sensory validation of functional snacks. Furthermore, adding functional ingredients such as resveratrol did not affect the sensory acceptability or organoleptic characteristics of the snacks. However, achieving commercial viability hinges on optimizing the formulation and bioactive compound concentrations, as well as ensuring consumer acceptance. Only by achieving this technical balance can sensory acceptability be maintained alongside enhanced nutritional value [2,32,41].

Table 6 shows the various processing technologies employed in the studies included in the review. The review identified various methods aimed at preserving the functional properties of snacks and functional foods. Extrusion and nano-encapsulation stand out as the most frequently used techniques, with both methods reported to improve the preservation of bioactive compounds. Compared to conventional drying methods, such as forced-air oven drying, technologies such as SCE-CO_2_ and LF were found not only to preserve, but also to improve the extraction efficiency and functional quality of snacks and functional foods.

## 4. Discussion

This review is the first study to systematically analyze the literature on functional snacks that could potentially improve mental health. Using search terms designed to be as exhaustive as possible, we identified 22 articles across three comprehensive databases that reported relevant research on functional snacks and foods. We also used a quality assessment instrument to ensure that the articles met specific standards. Functional foods are one of the most innovative areas of nutritional science [1,2]. Despite the exponential advances of food science and technology, the development of biologically supported functional snacks remains largely unexplored. Evidence suggests that this scarcity of literature reflects a methodological gap, whereby most research stops at physicochemical characterization, rather than a lack of commercial potential. This overlooks the link between innovation in the food matrix and its potential health impact. The most common snack types identified were fried extruded snacks [20], beverages [21], bread [22], bars [22,23], chocolates [11,25], and cookies [26]. The most frequently evaluated bioactive compounds and components were polyphenols (e.g., resveratrol, gallic acid and flavonoids/anthocyanins), essential macromolecules (e.g., proteins and fiber), omega-3 fatty acids, and probiotics. While antioxidant activity has been extensively explored as a functional property [20,21,22,23,24,26], snacks with neuroprotective properties remain scarce, and there is limited overlap among bioactive effects. Only one study reported antioxidant and anti-inflammatory properties in a functional beverage [21].

This review focused on functional foods as a new nutritional strategy that requires further research into their potential impact on mental health. This strategy is supported by food technology and sensory acceptability. Based on the findings, several observations emerged. Although the review included 22 articles, only eight focused on developing functional snacks. The remainder focused on the development of functional foods, and these were included because few studies addressed functional snacks. Table 2 shows the characteristics of the studies that focused on developing functional snacks. These were grouped by snack type and evaluation stage, as well as by the presence of biological evidence for antioxidant, anti-inflammatory, or other beneficial health properties. The table also shows the most relevant results of the studies.

However, few studies have evaluated the bioavailability and molecular pharmacokinetics of the bioactive compounds and food ingredients incorporated into functional snacks. While compound retention after extrusion is important, the available data remain insufficient. It is crucial to investigate their release and/or formation in the digestive tract, their biotransformation by the gut microbiota, and their permeability across the blood–brain barrier (BBB). Without data on the effective concentrations reaching target cells, the declared functionality remains theoretical, limiting its application in nutrition research [15,35,42,43]. The gut microbiota can biotransform dietary components into low-molecular-weight metabolites with specific physicochemical properties or transport mechanisms that may enable them to cross the BBB [1,44].

The composition and function of the gut microbiota can be affected by foods and their components, nutrients, or additives. Some bacterial strains are psychobiotic, meaning that they can influence brain function through the gut–brain axis. Their effects may involve short-chain fatty acids (SCFAs), neurotransmitters (e.g., GABA and serotonin), and indole derivatives, which participate in the gut-brain signaling. A recent systematic review identified the primary mechanisms of action of psychobiotic as regulating neurotransmitters (27.1%), modulating the gut microbiota (27.1%), producing SCFAs (16.9%), and controlling inflammatory responses (15.3%). These findings highlight the potential relevance of strains such as *Lactobacillus plantarum*, *Bifidobacterium breve*, and *Akkermansia muciniphila* [45]. Furthermore, dietary intake has been associated with emotional symptoms among medical students, though these associations are not necessarily causal. Lower consumption of unprocessed foods and higher intake of ultra-processed foods have been associated with behavioral profiles characterized by anxiety and depression. An exploratory cross-sectional study integrated into a previous cohort of Mexican medical students found that consuming more than three daily servings of cereals with added fats and sugars was associated with more than sevenfold higher odds of severe depressive symptoms. This finding highlights this dietary pattern as a potential factor associated with depressive symptoms [46].

Although the antioxidant properties of functional snacks have been widely explored, the limited integration of neuroprotective and anti-inflammatory properties means that they may not yet fulfill a comprehensive health promoting role. From a neurobiological perspective, nutritional imbalances resulting from poor diet and a lack of essential nutrients have been suggested as a potential contributors to systemic inflammatory responses, including the activation of pro-inflammatory pathways such as NF-κB and MAPK [13,47]. These pathways can lead to the release of pro-inflammatory cytokines, such as tumor necrosis factor alpha (TNF-α), interferon gamma (IFN-γ), and interleukins 1 and 6 (IL-1 and IL-6), as well as increased oxidative stress. These factors could adversely affect brain function [48,49]. Consequently, the evidence was limited to five articles that included in vitro and in vivo assessments. No clinical studies were found, so no human results were reported. Although antioxidant and anti-inflammatory properties have been described, further research is needed to confirm their biological and functional relevance and determine their potential contribution to functional nutrition.

Although functional nutrition overlaps to some extent with food innovation and technology, its primary importance lies in the bioactive compounds and components it provides, which contribute to the nutritional quality of snacks and other food products. While initial studies suggest potential benefits, further research is needed to confirm whether these components offer specific health benefits beyond basic nutrition. In an environment dominated by ultra-processed foods, developing functional snacks and foods represents a particularly significant contribution, particularly in the context of mental health. However, to consolidate this promising approach, it is essential to move beyond technological design and rigorously validate its biological effects and clinical relevance.

Therefore, the evidence indicates that integrating neurobiological and clinical validation, alongside precision nutrition [50], could be a viable strategy for advancing functional nutrition. In this context, targeted delivery systems, particularly for bioactive compounds with functional potential, could be a valuable tool for enriching snacks and functional foods, providing new nutritional approaches to support mental health. Furthermore, only one study progressed to the in vivo phase [27]. This highlights an opportunity for further validation and research, allowing techno-functional food innovation to contribute more significantly to population health through nutritional strategies. From a technological perspective, food processing engineering could help maintain or enhance food functionality.

Innovations in food technology, such as nanoencapsulation [20] and advanced methods such as freeze-drying [32] and HA-RFD [37], may help preserve bioactive compounds (*p* < 0.05), such as resveratrol. Nanoencapsulated resveratrol showed greater hypoglycemic effects (23.23% and 63.23%) and reduced adipogenesis (24.86–44.46%); in contrast, free resveratrol showed significantly lower inhibition (*p* < 0.05) at 18.93%. Furthermore, the reduced processing time and optimized thermal conditions of HA-RFD may contribute to the preservation of bioactive compounds, such as polyphenols (1496 mg GAE/100 g) and flavonoids (415 mg rutin/100 g). This is statistically significant (*p* < 0.05). These findings suggest that such technologies may help reduce oxidative degradation during processing, a factor that can affect the stability and shelf life of functional foods. The study using nanoencapsulated resveratrol reported lipid peroxidation inhibition exceeding 60% [20], supporting its antioxidant potential during processing and storage and its potential contribution to limiting oxidative deterioration [42]. The evidence indicates that technological innovation is an important component in the development of functional snacks, as processing conditions can influence whether bioactive compounds and their functional properties are preserved in the final product.

Finally, we identified another important observation regarding the inclusion of sensory evaluations in food development and technology. A key finding is the absence of sensory evaluations in most of the included studies. While various studies have shown that sample size and training levels can vary considerably, our findings revealed a significant disparity in sample size (*n*) across panels. Specifically, semi-trained panels of 15 to 20 individuals evaluated extruded snacks [20], whereas 50 untrained panelists assessed energy bars [23]. While a hedonic scale in sensory evaluation assesses attributes essential for acceptability, such as flavor, texture, aroma, and appearance, small sample sizes may limit the representativeness and robustness of acceptability findings, whereas the absence of trained panels may limit the detection of subtle sensory differences among formulations. This is particularly pertinent given the potential impact of fortification on sensory properties. For this reason, further research using appropriately designed consumer acceptability tests and when required, trained sensory panels, is recommended.

In terms of sensory attributes, the addition of compounds such as resveratrol (in its free or nanoencapsulated form) did not have a negative impact on flavor or overall acceptability, with scores remaining as high as 7.51 out of 9 [20]. While the studies concluded that fortification did not affect sensory acceptability, the evidence suggests that the small sample sizes and limited sensory evaluation designs provide limited support for this conclusion. Therefore, these results should be interpreted with caution. Sensory validation thus remains a significant gap in the current functional snack literature. For future formulation strategies, it is essential to incorporate these evaluations from the outset. The commercial success, acceptance, and optimal development of functional foods and snacks depend on the integration of food technology, the validation of their biological effects, and the assurance of sensory acceptability. This combined approach can support product functionality and consumer acceptance while strengthening the potential contribution of these products to health.

### Limitations of the Study

Despite the methods employed, this study has certain limitations. First, the number of studies on snacks designed to provide neuroprotective, anti-inflammatory, and antioxidant properties is limited. Therefore, it was important to include studies on functional foods in general to provide a broader technological background. While this inclusion was necessary to gain a better understanding of food technology innovation and the preservation of bioactive compounds, It introduced methodological heterogeneity that limits the direct generalization of the results to the specific design of functional snacks. A second, critical limitation of this systematic review was the level of scientific rigor of the identified literature. Most of the available studies focused on food development and characterization. We identified only one in vivo study and found no human clinical trials. Consequently, the benefits attributed to bioactive compounds used as functional ingredients in snacks are primarily derived from theoretical models or in vitro studies. The absence of biological and clinical validation means that these findings cannot be directly extrapolated to human health. Consequently, their relevance and efficacy must be interpreted with caution until sufficient clinical evidence becomes available. A further limitation was the scarcity of studies on functional snacks that included sensory evaluations, which limits the available evidence on consumer acceptance. Fourth, we acknowledge that we did not initially register this SLR in a formal protocol registry. Future studies should focus not only on developing and characterizing functional snacks/foods, but also on their biological validation through in vitro, in vivo, and clinical studies to evaluate the safety and efficacy of the developed functional snacks/foods. It is essential to thoroughly evaluate their technological properties alongside biological parameters such as antioxidant and anti-inflammatory activity, while ensuring optimal sensory acceptability. Finally, the search was restricted to freely available full-text articles, which represents an additional limitation. This methodological restriction was imposed to make the findings and evidence base easily accessible to the global academic community. However, the results should be interpreted with caution because studies available only through paid access were excluded.

## 5. Conclusions

This SLR reveals a significant discrepancy between technological innovation and functional validation. Of the initial pool of over 280,000 records, only 22 met the inclusion criteria. Of these, just 36.3% focused specifically on functional snacks, and only 12.5% of these studies biologically assessed neuroprotective properties biologically. Furthermore, food technologies such as nanoencapsulation and advanced drying (LF and HA-RFD) have been shown to preserve bioactive compounds more effectively. However, significant gaps remain in the evaluation of anti-inflammatory and neuroprotective properties, as well as in vivo, human, and sensory validation—key factors in assessing their potential health benefits and commercial feasibility. Further studies are needed to develop food matrices enriched with bioactive compounds with the potential to reduce oxidative stress and neuroinflammation. This would enable the evaluation of their potential role as adjunctive nutritional strategies to support mental health, as well as their level of consumer acceptance.

## Figures and Tables

**Table 1:** Eligibility criteria for selected studies.

Criteria	Inclusion Criteria	Exclusion Criteria
Type of study	Original studies involving the development and characterization of snacks/foods, including sensory evaluation.	Systematic reviews, meta-analyses, and literature reviews. In the case of clinical trials, the studies with participants outside the established age range.
Time period	Studies published between 2020 and 2025.	Studies that were not published between 2020 and 2025.
Language	Articles published in English or Spanish.	Articles that were not published in English or Spanish and did not have an English or Spanish translation.
Relevance	Articles that specifically investigated functional snacks/foods with neuroprotective, anti-inflammatory and antioxidant effects, using bioactive compounds as their main ingredients.	Articles that did not investigate functional snacks/foods with neuroprotective, anti-inflammatory, antioxidant, and other health-impacting properties were also excluded. Studies in which bioactive compounds were not the primary functional ingredients.
Subjects/Models	Studies using in silico, in vitro, and in vivo approaches, as well as clinical trials with participants of both sexes aged 18–26 years, related to functional snacks/foods. Defining the age range as 18–26 years reflects the need to assess a population with homogeneous consumption patterns and lifestyles that are characteristic of university life. Furthermore, this selection is an essential part of achieving the research project’s overall objective.	Studies on the neuroprotective effects, anti-inflammatory activity, and antioxidant capacity of bioactive compounds that were not related to snacks/functional foods.
Properties studied	Studies investigating bioactive compounds (e.g., polyphenols, flavonoids, amino acids, adaptogens, probiotics and omega-3 fatty acids) with neuroprotective effects, anti-inflammatory activity, and antioxidant capacity were included.	Studies that did not provide sufficient information on bioactive compounds (e.g., polyphenols, flavonoids, amino acids, adaptogens, probiotics, and omega-3 fatty acids) with neuroprotective, anti-inflammatory, or antioxidant properties.

**Table 2:** Characteristics of studies included in the review focused on the development of functional snacks.

Authors and Year	Functional Snack	Assessment Phase	Anti-Inflammatory/ Antioxidant Properties/ Other Properties	Key Findings
Ahmad and Gani, 2021 [20]	Fried extruded food	Food development and characterization	Antioxidant properties (40.78% and 61.85%), hypoglycemic properties (23.23% and 63.23%) and adipogenesis inhibition (NR 39.46%, 44.46% and 24.86%; FFR 18.93%)	Functional snacks contained higher levels of antioxidants, exhibited hypoglycemic properties, and inhibited adipogenesis more effectively than snacks that did not contain resveratrol or contained it in its free form.
Nguyen et al., 2025 [21]	Guava jelly drink	Food development and characterization	Antioxidant (EC_50_: DPPH 16.26 ± 1.18 mg/mL; FRAP 12.16 ± 0.74 mg/mL; ABTS 22.23 ± 0.55 mg/mL) and anti-inflammatory properties	The beverage exhibited antioxidant and anti-inflammatory properties, as well as the ability to suppress neuroregulatory enzymes (EC_50_: AchE 17.21 ± 1.62 mg/mL, MAO 71.42 ± 3.92 mg/mL, GABA-T 242.47 ± 7.27 mg/mL, COX-2 116.34 ± 11.83 mg/mL and GAD 1.23 ± 0.11 mg/mL).
Miranda et al., 2022 [22]	Functional low-calorie snacks (bread)	Food development and characterization	Antioxidant properties (IC_50_: ABTS 245.11 ± 4.66 µmol TE)	Compared with commercial snacks, the obtained snacks had a reduced calorie content (20–23%), a higher protein content (>60%), a higher fiber content, and a higher (>50%) antioxidant activity.
González-Calderón et al., 2021 [23]	Energy bars	Food development and characterization	Antioxidant properties (ORAC 9392.7 μmol TE/100 g)	Adding MV had a significant effect on the protein, lipid and fiber content, as well as the phenolic compound content and the antioxidant activity of the snacks.
Singh, Kumari and Chauhan, 2022 [24]	Functional bar for malnutrition	Food development and characterization	Antioxidant properties (DPPH %I: 48.50 ± 0.32, FRAP 3011.56 ± 0.96 µmol Fe(II)/100 g)	Adding oats and banana peel powder increased the functional bar’s content of protein, minerals, β-glucans, dietary fiber, essential amino acids, and phenolic compounds, as well as its antioxidant activity,
Faccinetto-Beltrán et al., 2022 [11]	Chocolate enriched	Food development and characterization. In vivo	Neuroprotective effects (microbial strains such as *Lactobacillus* (RT-PCR microbial quantification: tx 3.82 ± 0.17 vs. 3.51 ± 0.15 control) and *Bifidobacteria* (tx 3.82 ± 0.37 vs. 3.51 ± 0.16 control) were not affected, and there was a decrease in *Enterobacter* spp. (tx 3.75 ± 0.22 vs. 4.06 ± 0.29 control); the number of neurons increased in the CA1 (*p* < 0.05) and CA3 (*p* < 0.05) regions of the hippocampus.	The combination of chocolate with probiotics and omega-3 polyunsaturated fatty acids increased the number of neurons in the CA1 and CA3 regions of the hippocampus, providing protection against neurodegeneration and cognitive impairment. There was a marked improvement in learning by the fourth day in the mice that consumed the enriched chocolate, as demonstrated by short-term memory tests. Escape time was reduced by half compared with the control group (11.12 ± 0.87 s vs. 24.19 ± 5.1 s).
Medina-Mendoza et al., 2023 [25]	Dark chocolate enriched with Sacha inchi oil	Food development and characterization	Antioxidant properties (DPPH: 30.60 ± 0.13 to 53.10 ± 0.13 (μmol Trolox/g) and ABTS: 12.22 ± 0.06 to 22.21 ± 0.08 (μmol Trolox/g)).	The addition of Sauco by-products and Sacha inchi oil to dark chocolate, as well as increasing the conching time, resulted in significant changes (*p* < 0.05) in antioxidant activity, total phenol content (increasing from 22.15 to 24.65 mg GAE/g), rheology (increasing from 1.51 to 2.80 Pa.s), hardness (increasing from 3.35 to 6.98 N.) and particle size (increasing from 2.36 to 42.23 μm). The addition of Sauco by-products and Sacha inchi oil increased the antioxidant activity and total phenolic content of the dark chocolate, while Sacha inchi oil decreased its rheology and hardness.
Ali et al., 2023 [26]	Functional cookies	Food development and characterization	Antioxidant properties (average %I: DPPH 67.79 ± 4.50; ABTS 56.18 ± 15.85)	Using a combination of temperatures (OAT: 60 °C IAT: 150 °C), the maximum survival of the inoculated culture can be achieved, and the resulting powder could be used to develop functional cookies with improved sensory and biochemical characteristics.

NR: Nanoencapsulated resveratrol, FFR: Free-form resveratrol, %I: Percentage of inhibition, DPPH: 2,2-Diphenyl-1-picrylhydrazyl, FRAP: Ferric reducing antioxidant power, ABTS: 2,2′-Azino-bis (3-ethylbenzothiazoline-6-sulfonic acid, AchE: Acetylcholinesterase, MAO: Monoamine oxidase, GABA-T: GABA-transaminase, GAD: Glutamate decarboxylase, OAT: Outlet air temperature, and IAT: Inlet air temperature.

**Table 3:** Characteristics of complementary studies on functional foods.

Authors and Year	Functional Food	Assessment Phase	Anti-Inflammatory/ Antioxidant Properties/Other Properties	Key Findings
Chel-Guerrero et al., 2022 [27]	Habanero chili by-products. Extracted by supercritical fluid extraction (SFE), maceration (ME), and Soxhlet extraction (SOX).	Food development and characterization; in vitro and in vivo	The extract exhibited anti-inflammatory activities of 28.91 ± 1.61% and 24.60 ± 5.14%, as well as an antioxidant activity of 2.80 ± 0.0052 mM Trolox equivalent.	The extract obtained by SFE exhibited the most significant anti-inflammatory effect, whereas the extracts obtained by ME and SOX exhibited greater antioxidant activity and higher polyphenol content and polyphenol content.
Montserrat-de la Paz et al., 2021 [28]	Lupin protein hydrolysates (HPL) in Caco-2 and THP-1 cells.	Food development and characterization; in vitro	The hydrolysate exhibited antioxidant and anti-inflammatory activities, inhibiting 90% of COX-2, 22% of thrombin, and 50% of transglutaminase activity.	HPL crosses the intestinal monolayer (Caco-2 cells) and has an anti-inflammatory effect on macrophages. It reduces the levels of mRNA of pro-inflammatory cytokines, nitric oxide, and ROS.
Lara et al., 2022 [29]	Neuroprotective and resveratrol-modulating effects (RSV) in N2-A cells.	Food development and characterization; in vitro	Resveratrol reduced TNF levels from 3.2–5.2 g/mL to 0.5–1.2 g/mL, and stimulated IL-10 levels to reach 12–24 g/mL. It also exhibited antioxidant effects, reversing the marked increase in ROS induced by H_2_O_2_ (10.5% *v*/*v*), and reducing stress levels from peaks of 125–550 RLU/s to baseline values of 13–17 RLU/s.	RSV was found to have an anti-inflammatory and antioxidant effects on N2-A cells, which were dependent on the SIRT1 signaling pathway. Inhibiting SIRT with EX527 significantly (*p* < 0.001) attenuated protection by raising ROS to 55 RLU/s. However, AMPK blockade with dorsomorphin did not affect its activity.
Anorue et al., 2025 [30]	*Anacardium occidentale* (cashew) leaf extract.	Food development and characterization; in silico, in vitro, and in vivo.	The extract exhibited anti-inflammatory activity (maximum protein denaturation inhibition of 0.362 ± 0.002 at concentration of 0.15 mg/mL, and a significant, dose-dependent increase in protease inhibition capacity), as well as antioxidant activity (DPPH: IC50 of 49.36 µg/mL; FRAP: 0.25 mg/mL; TAC activity is dose-dependent (*p* ≤ 0.05); the highest NO scavenging ability was observed at 0.25 mg/mL).	The extract exhibited anti-inflammatory and antioxidant properties. This was achieved by suppressing the COX and LOX activity, thereby inhibiting the production of pro-inflammatory cytokines such as IL-6 and TNF-α (*p* < 0.05). In silico analysis revealed that flavones such as myricetin and catechin exhibited binding affinities comparable to those of drugs such as diclofenac and ibuprofen.
Alharaty and Ramaswamy 2025 [31]	Nutritionally enriched extruded matrix.	Food development and characterization	Antioxidant activity (%I): 72.30 ± 1.10 for samples enriched with RP + 15% MD, and 78.83 ± 0.90 for samples enriched with Mix + 5% MD).	The combination of RP and PP improved texture with firmness and tenacity increasing to 1503 g and 1822 g, respectively, as well as increasing anthocyanin content and antioxidant activity. Adding maltodextrin at a low concentration (5%) enhanced the antioxidant activity and anthocyanin retention by 98.6% in the mixture samples after extrusion and by 92.13% after drying.
Alam et al., 2023 [32]	Banana flours	Food development and characterization	The antioxidant activity of FD-Mehersagar flour was found to be 66.65 ± 1.54%.	The flour from the Mehersagar cultivar was found to have the highest retention of bioactive compounds. The quality parameters of banana flour make it suitable for use in the production of various gluten-free baked goods and flour-based food supplements. Among the drying methods, the Mehersagar cultivar flour showed the highest antioxidant activity.

The table presents the general characteristics of the additional studies included that focused on the development of functional foods. SFE: Extracted by supercritical fluid extraction, ME: Maceration, SOX: Soxhlet extraction, HPL: Lupine protein hydrolysates and RSV: Resveratrol, RLU/s: Relative light units per second, *v*/*v*: Volume/volume, TNF: Tumor necrosis factor, IL-10: Interleukin-10, RP: Rice protein, Mix: Protein mixtures, MD: Maltodextrin, %I: Percentage of inhibition.

**Table 5:** Characteristics of the selected studies that included sensory evaluation.

Authors and Year	Functional Snacks/Foods	Trained/Untrained Panel and Sample Size	Results
Ahmad and Gani, 2021 [20]	Fried, extruded food containing nanoencapsulated resveratrol.	A panel of 15–20 semi-trained panelists from the Department of Food Science and Technology at the University of Kashmir.	All the samples were equally well received by consumers. This suggested that the addition of resveratrol (free or encapsulated) did not affect the sensory characteristics of the product. Maximum scores of 7.51 ± 0.85 for flavor and 7.45 ± 0.44 for overall acceptability were obtained on a 9-point scale.
González-Calderon et al., 2021 [23]	Energy bars	50 untrained panelists	The panelists did not find any differences in sensory attributes between the samples (*p* > 0.05). The evaluation of sensory attributes yielded average scores ranging from 3.4 to 4.1 (on a scale of 0–5). Therefore, enrichment with plant samples did not affect sensory acceptability.

**Table 6:** Quantification of the included studies and the type of food technology used.

Articles Found	Food Technology Used to Preserve Functional Properties	Preserve Functional Properties?	References
1	Vacuum osmotic dehydration (VOD)	Yes	[36]
1	Carbon dioxide extraction (SCE-CO_2_ and SFE-CO_2_)	SCE-CO_2_ improved extraction efficiency	[37]
4	Extrusion	Yes	[20,31,33,40]
3	Nonencapsulated compounds	Yes, depending on the application objective	[20,34,35]
1	Co-encapsulated	Yes, depending on the application objective	[34]
1	Encapsulation	Yes, depending on the application objective	[38]
1	Rheological property	Yes	[39]
1	Spray drying	Yes	[26]
1	Freeze-drying (LF), cabinet drying (CD), microwave oven drying (MOD), and forced air oven drying (FOD)	Freeze-drying showed improved functional properties	[32]

VOD: Vacuum osmotic dehydration, SFE-CO_2_: Supercritical fluid extraction with carbon dioxide (CO_2_), LF: Freeze-drying, CD: Cabinet drying, MOD: Microwave oven drying, and FOD: Forced air oven drying.

## Data Availability

No new data were created or analyzed in this study. Data sharing is not applicable to this article.

## Supplementary Materials

The following supporting information can be downloaded at https://www.mdpi.com/article/10.3390/foods15183345/s1: Table S1. Search string; Table S2. Inclusion criteria; Table S3. Exclusion criteria; Table S4. Eligibility criteria for selected studies; Table S5. Candidate primary studies; Table S6. Selected primary studies; Table S7. Scale of quality questions applied to the included studies; Table S8. Quality criteria for the included studies; Table S9. Score for the quality criteria of the included studies; Table S10. Characteristics of primary studies focused on developing functional snacks; Table S11. Characteristics of complementary studies on functional foods; Table S12. Characteristics of studies using food technology to preserve functional properties; Table S13. Characteristics of the selected studies that included sensory evaluation; Table S14. Characteristics of the selected studies that included sensory evaluation; Table S15. Quantification of the included studies and the type of food technology used.

## Abbreviations

SRL	Systematic Literature Review
PUFAs	Polyunsaturated Fatty Acids
COVID-19	Coronavirus Disease 2019
PRISMA	Systematic Reviews and Meta-Analyses Statement
ATR-FTIR	Attenuated Total Reflectance-Fourier Transform Infrared Spectroscopy
SEM	Scanning Electron Microscopy
XRD	X-Ray Diffraction
DSC	Differential Scanning Calorimetry
BSE	Broccoli Sprout Extract
SDYP	Spray-Dried Yogurt Powder
OAT	Outlet Air Temperature
IAT	Inlet Air Temperature
AchE	Acetylcholinesterase
MAO	Monoamine Oxidase
GABA-T	GABA-Transaminase
GAD	Glutamate Decarboxylase
MV	Plant Mixtures
ω3	Omega-3
SFE	Supercritical Fluid Extraction
ME	Maceration
SOX	Soxhlet Extraction
HPL	Lupine Protein Hydrolysate
RSV	Resveratrol
VOD	Vacuum Osmotic Dehydration
HA-RFD	Radiofrequency-Assisted Hot Air Drying
HAD	Hot Air Dehydration
SCE-CO_2_	Subcritical Fluid Extraction with Carbon Dioxide (CO_2_)
SFE-CO_2_	Supercritical Fluid Extraction with Carbon Dioxide (CO_2_)
OFI	Opuntia Ficus-Indica
SPCS	Sorghum Protein Crocin System/Complex
FPCS	Foxtail Millet Crocin Protein System/Complex
RP	Rice Protein
PP	Pea Protein
MC	Moisture Content
AOA	Antioxidant Activity
ABA	Antibacterial Activity
β-lg	β-Lactoglobulin
BSG	Brewers’ Spent Grain
BBSG	Rye Brewers’ Spent Grain
LF	Lyophilized
CD	Cabinet Drying
MOD	Microwave Oven Drying
FOD	Forced Air Oven Drying
WHO	World Health Organization
FAO	Food and Agriculture Organization
IADSA	International Alliance of Dietary Supplement/Food Associations
